# Pure SH1 Guided-Wave Generation Method with Dual Periodic-Permanent-Magnet Electromagnetic Acoustic Transducers for Plates Inspection

**DOI:** 10.3390/s19133019

**Published:** 2019-07-09

**Authors:** Gongzhe Qiu, Xiaochun Song, Xu Zhang, Jun Tu, Tao Chen

**Affiliations:** Hubei Key Laboratory of Modern Manufacturing Quantity Engineering, School of Mechanical Engineering, Hubei University of Technology, Wuhan 430068, China

**Keywords:** dual PPM EMATs, SH1 guided-wave, positioning error, plate inspection

## Abstract

High frequency guided-waves offer a trade-off between the high sensitivity of local bulk ultrasonic thickness measurements and the large area scanning of lower frequency guided-waves, so it has been a growing interest for corrosion inspection with the dispersive SH1 mode. However, according to the dispersive curve, it is hard to generate the pure SH1 mode since the non-dispersive SH0 mode will be excited simultaneously. Thus, this paper investigates a transducer design method to generate a pure SH1 guided-wave, where the dual periodic-permanent-magnet electromagnetic acoustic transducers (PPM EMATs) are placed on exactly opposite positions either side of the plate symmetrically. The suppression effect for SH0 and the enhancement effect for SH1 of the dual PPM EMATs are mainly discussed by theoretical analysis and simulation analysis, and the influence of positioning errors of PPM EMATs placed on opposite sides of the plate on its performances are analyzed. Employing the proposed dual PPM EMATs, some experiments are performed to verify the reliability of finite element simulation. The results indicate that the dual PPM EMATs can suppress the SH0 mode and generate the pure SH1 mode effectively. Moreover, the longitudinal and lateral positioning errors can affect the dual PPM EMATs performances significantly.

## 1. Introduction

Nowadays the local bulk ultrasonic thickness measurements [1] and the low frequency guided-waves [2] have been proven to be efficient for corrosion damage inspection. The former has high detection sensitivity, small scanning region and long detection period, and the latter can scan large areas rapidly for big defects with lower detection sensitivity. As a compromise choice of the above two methods, the high frequency guided-waves is presented for corrosion inspection which can improve the detection sensitivity of guided-waves at the cost of scanning regions. The low frequency guided-waves conventionally try to suppress the dispersive behavior of guided-waves to acquire higher detection precision. However, the high frequency guided-waves evaluate the defects by the change of wave transit-time of dispersive guided-waves, and the dispersion behavior of guided-waves is a benefit to improve the detection sensitivity of high dispersive guided-waves [3]. In addition, for the distributed imaging of wall-loss defects, the scanning region of guided-waves should not be too large [4,5,6]. Therefore, the high frequency guided-waves has an increasing interest for corrosion damage inspection.

Currently, A0, S0, and SH1 mode can be used as highly dispersive guided-waves for corrosion inspection. Instanes G. [7] utilized the dispersion characteristic of A0 mode to inspect the corrosion defects in the circular pipe. Volker A. [8] monitored the circumferential thickness of pipes by measuring the arrival time and amplitude loss of high order circumferential passes. F. Jenot [9] evaluated the corrosion condition of the pipe by measuring the variation of group velocity of S0 mode. Compared with Lamb waves, SH guided-waves have such advantages as simpler dispersion property, less interference from surrounding media, and more convenient signal processing [10,11,12]. Uribe S.A. [13] and Nurmalia H. [14] investigated the mode conversion phenomenon when SH waves interact with the notch and found that SH0 mode can convert to SH1 mode as the inspected plate thickness increases abruptly more than two times. Nurmalia H. [15] studied the effect of the inclined angle of the wedge-shaped defect on propagation characteristics of the SH1 mode and found that the total reflection of SH1 mode can be observed if the inclination angle is small. Additionally, if the inclined angle becomes higher, the SH1 mode will convert to SH0 mode in the defect region and then convert back to SH1 mode after passing through the defect. Hirao M. [16] detected the dish-like defect in pipes by dispersive circumferential SH1 mode, compared with that of SH0 mode, the amplitude and the phase of SH1 mode is more sensitive to the defect. Since it is very difficult for the non-dispersive mode to detect the corrosion defect at a support location [17], Andruschak [18] utilized dispersive SH1 to inspect the corrosion defect at a support location. Richard [19] approximated pipes as planar waveguides and analyzed the detectability of corrosion damage with S0, SH0, and SH1 mode, respectively, and demonstrated the potential of high frequency short-range guided-waves detection technique. Accordingly, dispersive SH1 mode has the potential to be widely used in corrosion damage inspection.

According to the dispersive curve, the SH0 mode would be generated while exciting the dispersive SH1 mode. Thus, it is very difficult to generate pure dispersive SH1 mode while inspecting corrosion damages [20]. Alan C. Kubrusly et al. [21] proposed a method to generate pure SH1 guided-waves with dual PPM EMATs, but they only explain the phenomenon from the linear superposition point of view. In this paper, the theoretical model based on the transduction mechanism and solid mechanics is established. Section 2 presents the theoretical model of generating and receiving pure SH1 mode based on the transduction equations. Section 3 describes the characters of the magnetic field generated by single and double permanent magnet arrays, respectively, establish a theoretical model based on solid mechanics to discuss the influence of the lateral and longitudinal position errors of dual PPM EMATs on its performance. Section 4 reports and analyzes the experimental measurement results of the proposed dual PPM EMATs.

## 2. Transduction Mechanism

### 2.1. Excitation Mechanism

Supposed that free charge does not exist and the effect of displacement current is ignored in the system consisting of EMAT coils and the non-ferromagnetic specimen, the dynamic magnetic field equations of pulsed eddy current can be deduced based on Ampere’s Laws and Faraday’s Laws.

(1) $$\frac{1}{\mu }\nabla^{2}A-\sigma \frac{\partial A}{\partial t}+\frac{1}{S}\overset{\;}{\underset{S}{\iint }}\sigma \frac{\partial A}{\partial t}ds=-\frac{i}{S}$$

where A is magnetic vector potential, σ is conductivity, S is the cross-sectional area of the coil, and i is the total current in the coil.

The eddy current density $J_{e}$ in the coil and the non-ferromagnetic specimen gives

(2) $$J_{e}=-\sigma \frac{\partial A}{\partial t}$$

Based on the definition of Lorentz force, the Lorentz force $f_{L}$ in the nonferromagnetic specimen is

(3) $$f_{L}=B_{0}\times J_{e}$$

where $B_{0}$ is the magnetic flux density of the static bias magnetic field. Generally, the dynamic magnetic field is ignored as it is much weaker than the static one when the excitation current is not high [22].

Assuming that the non-ferromagnetic specimen is isotropic, linear elastic and continuous, the equation of particle motion in the non-ferromagnetic specimen under the Lorentz force $f_{L}$ is

(4) $$G\nabla^{2}u+\left(G+\kappa \right)\nabla \left(\nabla \cdot u\right)+f_{L}=\rho \frac{\partial^{2}u}{\partial t^{2}}$$

where G and κ are the Lame constant, u is the particle displacement matrix, ρ is the density of the specimen.

**Figure 1:**
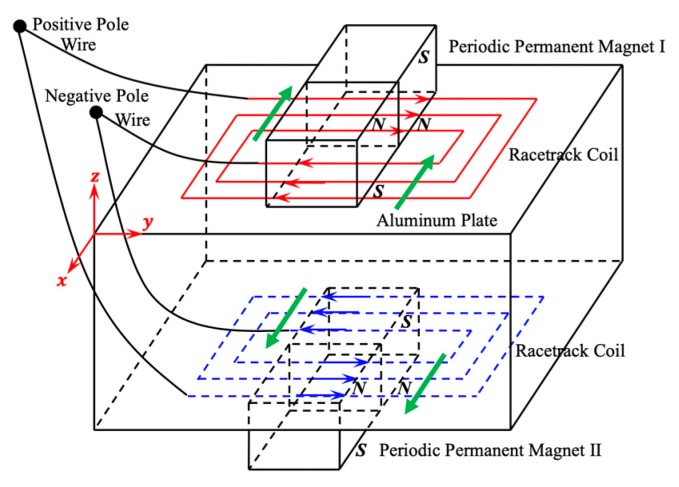
Schematic of the SH1 wave excited by dual PPM EMATs.

As shown in Figure 1, two racetrack coils with opposite spiral direction are placed on the exact opposite sides of the aluminum plate symmetrically. In order to install magnets and enhance the vertical bias magnetic field effectively, magnetic poles of PPM on the two sides of the aluminum plate are in the same direction. The red and blue arrows in Figure 1 represent the direction of external current in the upper and lower racetrack coils respectively. The green arrows represent the direction of particle vibration in the aluminum plate. It can be seen that the direction of loaded current in the upper and lower coil are opposite, namely

(5) $$J_{eup}=-J_{edown}$$

where $J_{eup}$ and $J_{edown}$ are the eddy current density on the upper and lower surfaces of aluminum plate, respectively.

Substituting Equation (5) into Equations (3) and (4), gives

(6) $$u_{xup}=-u_{xdown}$$

where $u_{xup}$ and $u_{xdown}$ are the tangential particle displacement on the upper and lower sides of aluminum plate respectively. Equation (6) illustrates that the tangential particle displacement on the two sides are opposite, namely the generation of symmetric mode can be suppressed effectively by dual PPM EMATs.

### 2.2. Receiving Mechanism

When the guided-wave in the aluminum plate travels along the area covered by coils, under the external bias magnetic field, the alternating current will be induced in the coil by the vibration of charged particles, which can generate dynamic magnetic field in and around the aluminum plate. The receiving coil in the dynamic magnetic field will generate induced voltage. In general, if the receiving coil is open and its total current is zero, the control equation satisfied by the receiving coil and the tested sample is

(7) $$-\frac{1}{\mu }\nabla^{2}A+\sigma \frac{\partial A}{\partial t}-\frac{\sigma }{S}\frac{\partial }{\partial t}\overset{\;}{\underset{S}{\iint }}Ads=J_{L}$$

where $J_{L}$ is dynamic current density, $J_{L}=\sigma v\times B_{0}$, v is the velocity of the charged particles. Additionally, the electromotive force at some point in the receiving coil can be calculated by the line integral for electric field intensity.

(8) $$V_{pout}=\int_{l}-\frac{\partial A}{\partial t}\cdot dl$$

The output voltage can be obtained by averaging the electromotive force in the receiving coil.

(9) $$V_{out}=\frac{\iint_{S}^{\;}V_{pout}dS}{\iint_{S}^{\;}dS}$$

**Figure 2:**
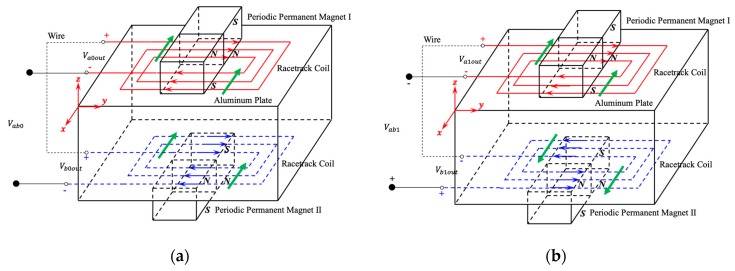
Schematic of the SH wave received by dual PPM EMATs: (**a**) SH0 waves; (**b**) SH1 waves.

Figure 2a shows the receiving process of SH0 waves detected by dual PPM EMATs, and green arrows represent the vibration direction of particles on the upper and lower surfaces of the aluminum plate, red and blue arrows represent the direction of induced current in the upper and lower racetrack coils respectively. As shown in Figure 2a, the vibration direction of particles on the two opposite sides are the same because SH0 waves is the symmetric mode, namely

(10) $$v_{0up}=v_{0down}$$

where $v_{0up}$ and $v_{0down}$ are the vibration velocity of particles on the upper and lower surfaces of the aluminum plate.

Substituting Equation (10) into $J_{L}=\sigma v\times B_{0}$, gives:

(11) $$J_{L0up}=J_{L0down}$$

where $J_{L0up}$ and $J_{L0down}$ are the density of dynamic current on the upper and lower surfaces of the aluminum plate, respectively.

Substituting Equation (11) into Equations (7)–(9), gives:

(12) $$V_{a0out}=V_{b0out}$$

where $V_{a0out}$ and $V_{b0out}$ are the output voltage of the upper and lower transducers, respectively. Additionally, the output voltage $V_{ab0}$ under SH0 waves is

(13) $$V_{ab0}=V_{a0out}-V_{b0out}=0$$

Equation (13) shows that when the SH0 waves reach the receiving coil of dual PPM EMATs, the directions of dynamic current in the upper and lower racetrack coils are the same due to the same vibration direction of particles on the two sides, and the output voltage of the receiving coil is zero. Thus, the receiving dual PPM EMATs can filter out the voltage signal generated by SH0 mode effectively, and improve the SNR of receiving signal.

Similarly, Figure 2b is the receiving process of SH1 waves detected by dual PPM EMATs, and green arrows represent the vibration direction of particles on the upper and lower surfaces of the aluminum plate, red and blue arrows represent the induced current in the upper and lower racetrack coils, respectively. However, as shown in Figure 2b, the vibration direction of particles on the upper and lower surfaces are opposite because SH1 waves is the anti-symmetric mode, that is

(14) $$v_{1up}=-v_{1down}$$

where $v_{1up}$ and $v_{1down}$ are the vibration velocity of particles on the upper and lower surfaces of the aluminum plate.

Substituting Equation (14) into $J_{L}=\sigma v\times B_{0}$ gives:

(15) $$J_{L1up}=-J_{L0down}\;$$

where $J_{L0up}$ and $J_{L0down}$ are the density of dynamic current on the upper and lower surfaces of the aluminum plate.

Substituting Equation (15) into Equations (7)–(9) gives:

(16) $$V_{a1out}=-V_{b1out}$$

where $V_{a1out}$ and $V_{b1out}$ are the output voltage of the upper and lower transducers, respectively. Additionally, the output voltage $V_{ab1}$ under SH1 waves is

(17) $$V_{ab1}=V_{a1out}-V_{b1out}=2V_{a1out}$$

Equation (17) shows that when the SH1 waves reach the receiving coil of dual PPM EMATs, the directions of dynamic current in the two racetrack coils are the opposite due to the opposite vibration directions of particles on the upper and lower surface, and the output voltage of the receiving coil is doubled. Thus, in the receiving process of the guided-waves, the dual PPM EMATs can enhance the voltage signal generated by SH1 mode effectively. The dual PPM EMATs can suppress the symmetric mode in the generation of SH1 mode, and enhance the anti-symmetric mode in the receiving of SH1 mode.

## 3. Simulation Analysis

The distribution characteristics of static magnetic field of conventional PPM EMATs and dual PPM EMATs proposed in this paper are firstly investigated, and the suppression for SH0 mode are analyzed by comparing the excitation signal excited by two different PPM EMATs, respectively. Additionally, the effects of lateral and longitudinal position errors of the PPM EMATs on the SNR of excitation signals are discussed.

### 3.1. Distribution of Static Magnetic Field Induced by Conventional PPM EMATs and Dual PPM EMATs

**Figure 3:**
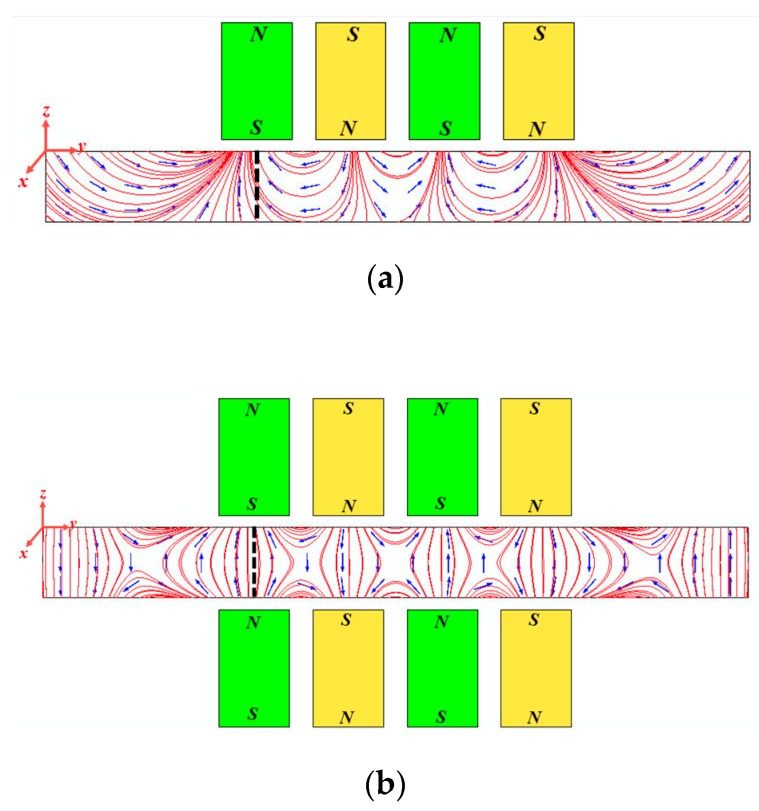
Bias magnetic field distribution: (**a**) single PPM arrays; (**b**) dual PPM arrays.

Static bias magnetic field is a major factor affecting the transducing efficiency of EMATs [23]. Optimizing the geometric structure and dimension parameters of EMATs can enhance the intensity [24,25] and uniformity [26,27] of bias magnetic field, and improve the transducing efficiency and detection sensitivity. Dutton [28] enhanced the intensity and uniformity of bias magnetic field significantly using the compressed magnetic field generated by a couple of permanent magnets. Figure 3a,b show the magnetic distribution in plates under the single and dual PPM arrays, and red solid lines represent the magnetic lines of force, blue arrows represent the direction of the static magnetic field. In Figure 3, each permanent magnet has 3 mm width and 5 mm thickness, the horizontal spacing is 1 mm, and the magnetic flux density is 1.5 T. The lift-off between the aluminum plate and permanent magnetic arrays is 0.5 mm. The aluminum plate is 30 mm long and 3 mm thick, and the density $\rho =2700\;\mathrm{kg}/m^{3}$, relative permeability $\mu_{r}=1$ and conductivity $\gamma =3.774\times 10^{7}\left[S/m\right]$. The excitation of SH waves mainly depends on the coupling of induced eddy current and vertical bias magnetic field Figure 3 shows that the vertical distribution of magnetic lines generated by dual PPM arrays is better than that by single PPM arrays clearly.

**Figure 4:**
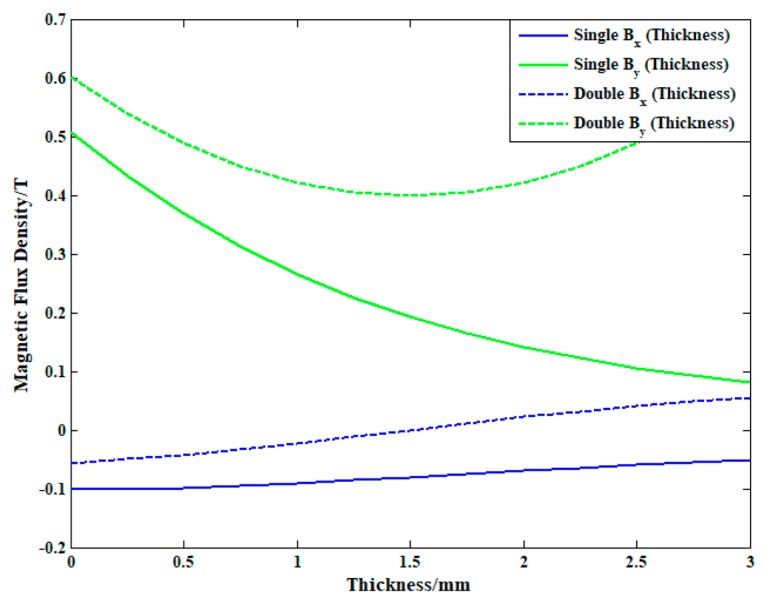
Distribution of the magnetic flux density in the aluminum plate

In order to analyze the distribution uniformity of the static magnetic fields quantitatively, the vertical and horizontal components of the magnetic flux density along the thickness of aluminum plate are extracted. Additionally, Figure 4 shows the vertical and horizontal components of the magnetic flux density along the thickness of aluminum plate (i.e., black dashed line in Figure 3), solid lines represent the vertical and horizontal components of the magnetic flux density generated by single PPM arrays, dashed lines represent that of dual PPM arrays. Since the skin depth of the aluminum plate under 700 kHz is $\Delta =\sqrt{2/\left(\omega \mu \gamma \right)}=0.0979\;\mathrm{mm}$, the magnetic flux density in the depth of 0.1 mm is selected as a reference. From Figure 4, it is found that the vertical component of magnetic flux density of single PPM arrays $B_{ys}$ = 0.48 T, and that of dual PPM arrays is $B_{yD}$ = 0.58 T, increasing by 21%. Additionally, the horizontal component of magnetic flux density of single PPM arrays $B_{xs}$ = 0.1 T, and that of dual PPM arrays is $B_{xD}$ = 0.05 T, decreasing by 50%. That is to say, the dual PPM arrays can increase the vertical component and reduce the horizontal component of magnetic flux density at the skin depth, which improves the transducing efficiency. In addition, from the upper surface to the middle plane of the aluminum plate, the vertical component of magnetic flux density of single PPM arrays $B_{ys}$ decreases from 0.5 T to 0.2 T with 80% decreasing, and that of dual PPM arrays $B_{yD}$ decreases from 0.6 T to 0.4 T with 33% decreasing. Obviously, the vertical component of magnetic field generated by dual PPM arrays distributes more uniform than that of by single PPM arrays, which is beneficial to suppress the noise and improve the SNR of excitation signal.

### 3.2. Signals Excited by Three PPM EMATs with Different Configurations

**Figure 5:**
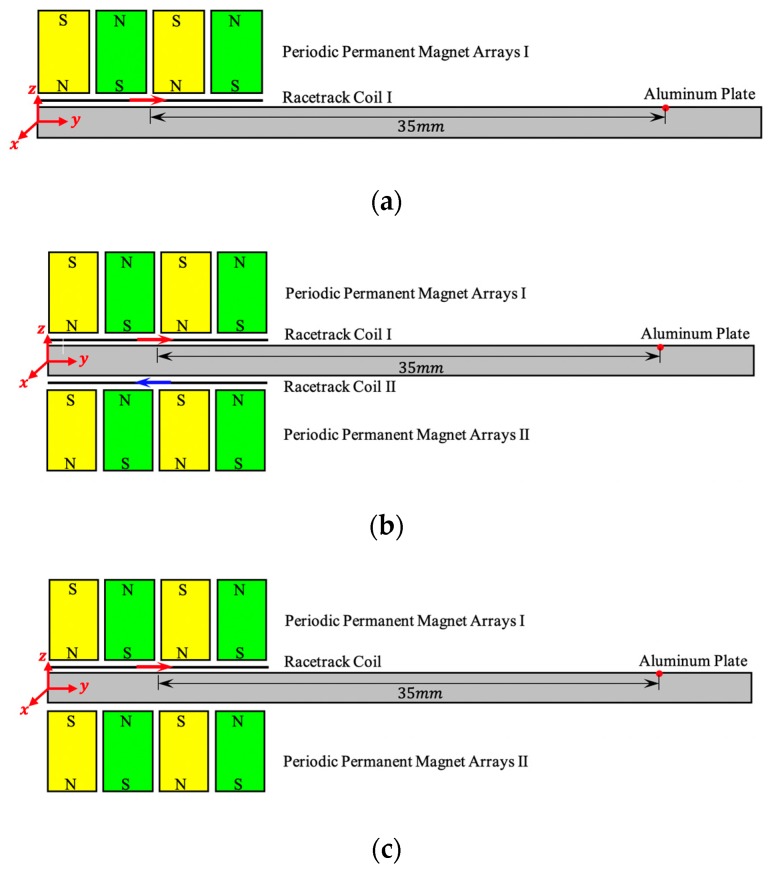
Three different PPM EMATs configurations: (**a**) Single PPM arrays and single coil; (**b**) dual PPM arrays and single coil; and (**c**) dual PPM arrays and dual coils.

Figure 5a–c shows three different configurations of PPM EMATs to generate SH waves. The capacity to suppress symmetric modes of conventional PPM EMATs and dual PPM EMATs are simulated. To decrease the computation cost and ensure the wave packets separation from SH0 mode and SH1 mode absolutely, the length of aluminum plate in simulation model is set as 50 mm. The transducers are located at the left side of the aluminum plate, and the vibration displacement amplitude in the x direction is extracted with 35 mm distance from the transducer center.

The 3D simulation models for three different PPM EMATs shown in Figure 5 have been developed based on the COMSOL Multiphysics software. Table 1 shows the geometrical parameters of PPM EMATs and the physical property of the Aluminum plate in the FEM. In order to avoid the disturbance of reflected waves, the aluminum plate is surrounded by a layer of absorbing layers with increasing damping (ALID) [29]. Additionally, the aluminum plate is divided into 15 cubic grids [30] per each wavelength along and perpendicular to the direction of SH1 waves propagation and into 5 in the thickness direction to balance the computation cost between the precision. Therefore, the total number of elements is about 17220. The iteration step time is set as $1\times 10^{-8}$ s to satisfy the stability criterion as $\Delta t<0.8\Delta x/C_{max}$, for which Δx is the element size and $C_{max}$ is the velocity of wave that travels fastest through the material [31].The excitation signal is the sinusoid modulated by five cycles of Hamming windows. Only one exciting unit of the dual probes is established and the racetrack coil is simplified as the straight wire to reduce the iteration time with satisfactory accuracy.

**Figure 6:**
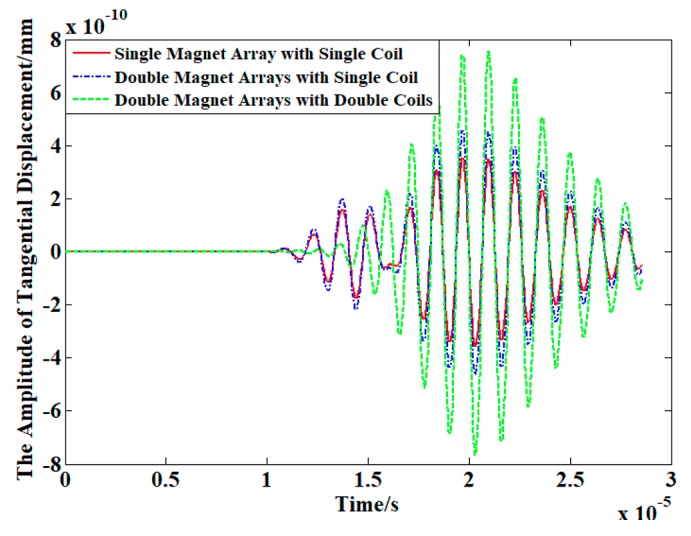
Exciting signals of three different configurations of PPM EMATs.

Figure 6 shows the signal excited by three different configurations of PPM EMATs shown in Figure 5. Additionally, the red solid line, blue dash-dotted line and green dashed line in Figure 6 correspond to the configurations shown in Figure 5a–c, respectively. As shown in Figure 6, the amplitude of blue signals is 20% higher than that of red signals because the dual PPM arrays enhance the vertical bias magnetic field. Additionally, compared to the red signals, the amplitude of green signals is doubled due to the linear superposition effect of dual PPM EMATs. Moreover, wave packet separation phenomenon between SH0 mode and SH1 mode can be seen in red signals and blue signals, but only pure SH1 wave packet exists in green signals. In conclusion, compared with the conventional one shown in Figure 5a,b, the dual PPM EMATs shown in Figure 5c can not only suppress symmetric modes effectively, but also enhance non-symmetric modes significantly.

### 3.3. Effects of Position Errors of Dual PPM EMATs on Its Performances

**Figure 7:**
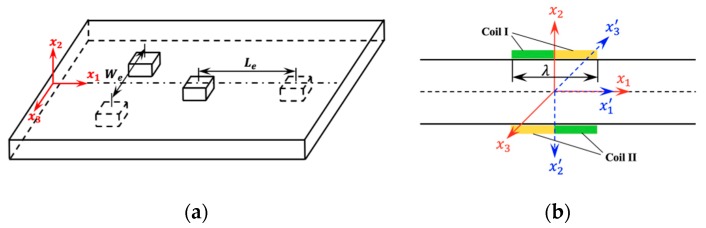
The coordinate system (**a**) Schematic of positioning errors in dual PPM EMATs. (**b**) The superposition effect of dual probes.

While dual PPM EMATs can suppress the generation of symmetric modes and enhance the SH1 waves effectively, it is essential to ensure the exact symmetry of PPM EMATs placed on each side of the inspected plate in applications. Thus, the effects of position errors on the performance of dual PPM EMATs are discussed in this section. As shown in Figure 7a, the position error of dual PPM EMATs can be classified as lateral error $W_{e}$ and longitudinal error $L_{e}$. In order to simplify the model and decrease the computation cost, only one exciting unit of dual probes, which includes one exciting unit of the upper and lower probe is built in FEM model.

In order to investigate the effect of longitudinal error $L_{e}$ on the performance of dual probes, the $x_{1}-x_{2}-x_{3}$ coordinate system for the upper transducer and $x_{1}^{\prime }-x_{2}^{\prime }-x_{3}^{\prime }$ for the lower transducer are established, as shown in Figure 7b. Additionally, SH waves travel in the $x_{1}\left(\mathrm{or}\;x_{1}^{\prime }\right)$ direction and the particles vibrate in the $x_{3}\left(\mathrm{or}\;x_{3}^{\prime }\right)$ direction. The transformation matrix between two coordinate systems is:

$$\left[\begin{array}{c} x_{1}\\ x_{2}\\ x_{3} \end{array}\right]=\left[\begin{array}{ccc} 1 & 0 & 0\\ 0 & -1 & 0\\ 0 & 0 & -1 \end{array}\right]\left[\begin{array}{c} x_{1}^{\prime }\\ x_{2}^{\prime }\\ x_{3}^{\prime } \end{array}\right]$$

For any isotropic medium, Navier’s displacement equations of motion must be satisfied:

(18) $$\mu \nabla^{2}u\left(x,t\right)+\left(G+\kappa \right)\nabla \nabla \cdot u\left(x,t\right)=\rho \frac{\partial^{2}u\left(x,t\right)}{\partial t^{2}}\;$$

For SH modes, an $x_{3}$ component of the particle displacement vector only exists, that is, $u_{1}\left(x,t\right)=u_{2}\left(x,t\right)=0$. The fact that $u_{3}$ is independent of $x_{3}$ means the wavefronts are infinitely extended in the $x_{3}$ direction. Then, the Equation (18) can be simplified as

(19) $$\frac{\partial^{2}u_{3}}{\partial x_{1}^{2}}+\frac{\partial^{2}u_{3}}{\partial x_{2}^{2}}=\frac{1}{c_{T}^{2}}\frac{\partial^{2}u_{3}}{\partial t^{2}}\;$$

where $c_{T}^{2}=\kappa /\rho$.

In the $x_{1}-x_{2}-x_{3}$ coordinate system, the form of $u_{3}$ is specified as

(20) $$u_{3}\left(x_{1},x_{2},t\right)=f\left(x_{2}\right)e^{i\left(kx_{1}-\omega t\right)}$$

and in the similar way, the form of $u_{3}^{\prime }$ in the $x_{1}^{\prime }-x_{2}^{\prime }-x_{3}^{\prime }$ coordinate system is

(21) $$u_{3}^{\prime }\left(x_{1}^{\prime },x_{2}^{\prime },t\right)=-f\left(x_{2}^{\prime }\right)e^{i\left[k(x_{1}^{\prime }+\Delta x\right)-\omega t]}\;$$

where k is the wavenumber of the mode ($k=\omega /c_{p}=2\pi /\lambda$) and ω represents circular frequency.

Substituting Equation (20) into Equation (19) results in

$$f\left(x_{2}\right)=\left[Asin\left(qx_{2}\right)+Bcos\left(qx_{2}\right)\right]$$

Then the displacement field $u_{3}\left(x_{1},x_{2},t\right)$ generated by the upper probe can be expressed as

(22) $$u_{3}\left(x_{1},x_{2},t\right)=\left[Asin\left(qx_{2}\right)+Bcos\left(qx_{2}\right)\right]e^{i\left(kx_{1}-\omega t\right)}$$

Substituting Equation (21) into Equation (19) results in

$$f\left(x_{2}^{\prime }\right)=\left[Asin\left(qx_{2}^{\prime }\right)+Bcos\left(qx_{2}^{\prime }\right)\right]$$

Then the displacement field $u_{3}^{\prime }\left(x_{1}^{\prime },x_{2}^{\prime },t\right)$ generated by the lower probe can be expressed as

$$u_{3}^{\prime }\left(x_{1}^{\prime },x_{2}^{\prime },t\right)=-\left[Asin\left(qx_{2}^{\prime }\right)+Bcos\left(qx_{2}^{\prime }\right)\right]e^{i\left[k(x_{1}^{\prime }+\Delta x\right)-\omega t]}$$

Expressing $u_{3}^{\prime }\left(x_{1}^{\prime },x_{2}^{\prime },t\right)$ in the $x_{1}-x_{2}-x_{3}$ coordinate system results in

(23) $$u_{3}^{\prime }\left(x_{1},x_{2},t\right)=-\left[Asin\left(-qx_{2}\right)+Bcos\left(-qx_{2}\right)\right]e^{i\left[k(x_{1}+\Delta x\right)-\omega t]}$$

$$=\left[Asin\left(qx_{2}\right)-Bcos\left(-qx_{2}\right)\right]e^{i\left(kx_{1}-\omega t\right)}e^{i\left(k\Delta x\right)}$$

$$=\left[ADsin\left(qx_{2}\right)-BDcos\left(qx_{2}\right)\right]e^{i\left[kx_{1}-\omega t\right]}$$

where

(24) $$D=e^{i\left(k\Delta x\right)}=e^{i\left(2\pi \Delta x/\lambda \right)}=\cos\left(2\pi \Delta x/\lambda \right)+i\sin\left(2\pi \Delta x/\lambda \right)$$

Based on the linear superposition effect, the displacement field generated by the dual probes can be expressed as:

$$u_{3total}\left(x_{1},x_{2},t\right)=u_{3}\left(x_{1},x_{2},t\right)+u_{3}^{\prime }\left(x_{1},x_{2},t\right)$$

$$=\left[\left(1+D\right)Asin\left(qx_{2}\right)+\left(1-D\right)BDcos\left(qx_{2}\right)\right]e^{i\left[kx_{1}-\omega t\right]}$$

Then, the amplitude of SH1 mode is:

(25) $$H_{SH1}=\sqrt{{\left[1-\cos\left(2\pi \Delta x/\lambda \right)\right]}^{2}+{\left[\sin\left(2\pi \Delta x/\lambda \right)\right]}^{2}}A=2Acos\left(\pi \Delta x/\lambda \right)$$

Additionally, in the similar way, the amplitude of SH0 mode is:

(26) $$H_{SH0}=\sqrt{{\left[1-\cos\left(2\pi \Delta x/\lambda \right)\right]}^{2}+{\left[\sin\left(2\pi \Delta x/\lambda \right)\right]}^{2}}B=2Bsin\left(\pi \Delta x/\lambda \right)\;$$

**Figure 8:**
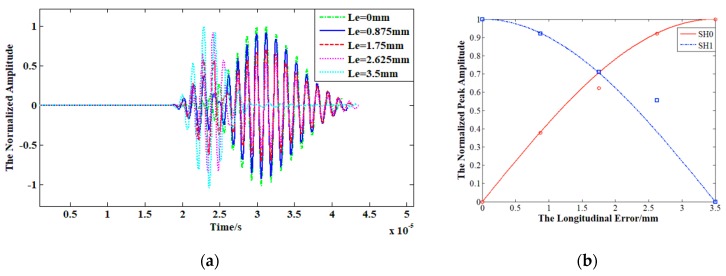
The effect of longitudinal error Le on the performance of dual probes: (**a**) The waveforms of SH0 and SH1 with different Le. (**b**) The normalized peak amplitude of SH0 and SH1 with different Le.

Figure 8 shows the effect of longitudinal error Le on the performance of dual probes. Figure 8a gives the simulation waveforms at the point 60 mm from the exciting transducer with different Le. The amplitude is normalized by the maximum of all signals. The red solid line and blue dash line in Figure 8b represent the theoretical value of SH0 and SH1 calculated by Equations (25) and (26), respectively. Additionally, the blue squares and red circles stem from the simulation results of SH0 and SH1, respectively. As shown in Figure 8b, the theoretical values and simulation results agree well. It can be concluded that the amplitude of SH1 decrease by cosine and that of SH0 increase sinusoidally with the longitudinal error $L_{e}$ increasing from 0 to half of the wavelength λ/2.

When the longitudinal error $L_{e}$ is zero and the lateral error $W_{e}$ is within one wavelength, the simulation result shows that dual probes can still suppress SH0 mode effectively and generate pure SH1 mode. The reason may be that the wavefronts are infinitely extended in the $x_{3}$ direction, as indicated by Equations (20) and (21), so the transverse vibration of particles can be considered uniform in the $x_{3}$ direction when $W_{e}$ is within one wavelength.

## 4. Experimental Analysis

**Figure 9:**
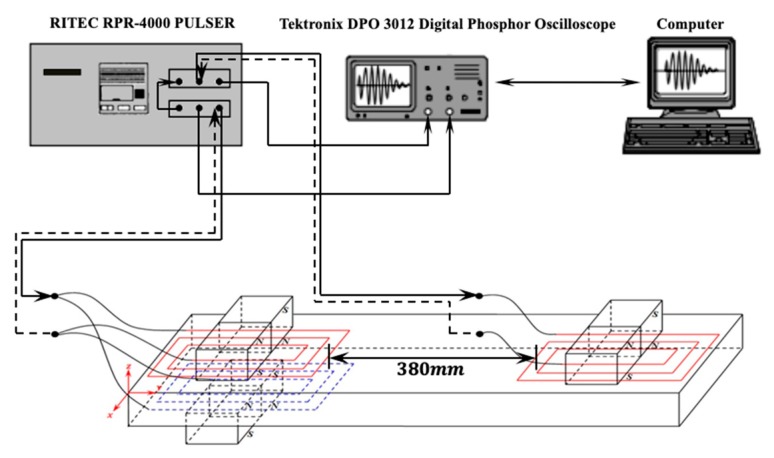
Schematic of experimental setups.

The theoretical analysis and simulation analysis indicate that dual PPM EMATs can suppress SH0 mode and enhance SH1 mode in the generation and reception process of SH waves. In order to verify the above results, an experimental setup, shown in Figure 9, is developed under laboratory conditions. The experimental setup includes a RITEC RPR-4000 Pulser/Receiver, a Tektronix DPO 3012 Digital Phosphor Oscilloscope (Shanghai, China), an aluminum plate with 3 mm-thickness and the PPM EMATs. In order to prevent echo interference reflected from the end of the aluminum plate, the exciting and receiving transducers are placed closed to the left and right edges of the aluminum plate respectively, which reduce the wave path-difference between the direct waves and the reflected echo from the left end. As the length of aluminum plate and racetrack coil are 500 mm and 60 mm, it can be calculated that the distance between exciting and receiving transducers is 380 mm. Additionally, three cases with different transducers configuration such as single exciting and receiving PPM EMATs (I), single exciting and dual receiving PPM EMATs (II), dual exciting and single receiving PPM EMATs (III), are discussed in the experimental platform shown in Figure 9. Comparing with case I and case II, the suppression effect for SH0 and the enhancement effect for SH1 of the dual PPM EMATs in the receiving process of SH waves can be analyzed. Similarly, the suppression effect for SH0 and the enhancement effect for SH1 of the dual PPM EMATs in the exciting process of SH waves can be discussed by comparing with case I and case III.

And the parameters and values of PPM EMATs used in the experiment are shown in Table 2. Since the frequency of excitation current is 700 kHz and the phase velocity of SH1 mode in the aluminum plate is 4900 m/s, the wavelength of SH1 mode can be calculated as 7 mm.

**Figure 10:**
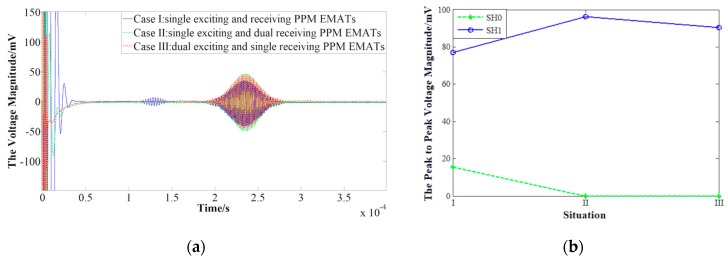
Amplitude of receiving signal with three different PPM EMATs configurations. (**a**) The original waveform. (**b**) The peak to peak voltage magnitude.

Figure 10 shows the amplitude of receiving signals with different PPM EMATs configurations. The time of flight it takes to travel through 380 mm indicates that the smaller waveform in Figure 10a is SH0 waves (1.2$\;\times 10^{-4}\;s$) and the other is SH1 waves (1.8 $\times 10^{-4}\;s$). Compared with case I and case II, it is found that the amplitude of SH0 wave decreases from 18 mV to zero, and the amplitude of SH1 waves increases from 77 mV to 97 mV. That is to say, SH0 mode is suppressed effectively and SH1 mode can be enhanced significantly if the dual PPM EMATs are employed to receive SH waves. Similarly, by comparison of case I and case III, it can be seen that the suppression effect for SH0 and the enhancement effect for SH1 are also effective while using the dual PPM EMATs to excite SH waves.

**Figure 11:**
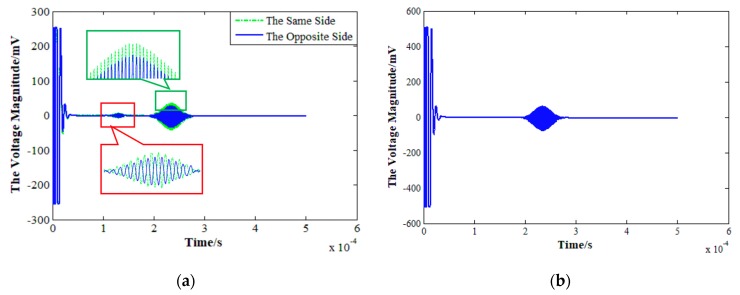
SH wave signals: (**a**) Global graph and local enlarged detail of SH0 and SH1; (**b**) Positive superposition signals.

The case II will be further investigated to reveal the superposition mechanism of SH0 waves and SH1 waves. Figure 11a shows the received signals by the single PPM EMATs placed on the upper and lower surface of the plate respectively while the single exciting PPM EMATs fixed on the upper surface. The green dashed line represents signals received when the exciting and receiving transducers are placed on the same side of the aluminum plate, the blue solid line represents signals received, while the exciting and receiving transducers placed on the different side of the plate. Figure 11b gives the positive superposition signal received by the upper and lower transducers.

As shown in Figure 11a, the signal amplitude is slightly higher when the exciting and receiving transducers are placed on the same side. The time of flight it takes to travel through 380 mm indicates that the smaller waveform in Figure 11a is SH0 waves ($1.2\times 10^{-4}\;s$) and the other is SH1 waves ($1.8\times \;10^{-4}\;s$). It should be noticed that the phases of SH0 mode received by the upper and lower probes are opposite, and SH1 mode has the same phases. Compared with Figure 11a, the SH0 waveform is eliminated in Figure 11b and the peak-to-peak voltage magnitude increases from 77 mV to 137 mV. It can be concluded that the SH0 mode can be suppressed and the SH1 mode is enhanced if superposing the signal received by the upper and lower transducers, which agrees with the theoretical and simulation results well. Additionally, the peak to peak voltage magnitude of waveform shown in Case II of Figure 10a is only 96 mV, which is much smaller than that shown in Figure 11b. The discrepancy may result from the energy loss at the connector for upper and lower probes.

## 5. Conclusions

A method to generate pure SH1 guided-waves is investigated in this paper. The SH0 suppression effect and SH1 enhancement effect of dual PPM EMATs are mainly analyzed. The effect of position errors of the PPM EMATs on its performances is also discussed. The simulation and experimental results demonstrate that the dual PPM EMATs, placed on the upper and lower sides of the inspected plates symmetrically, can suppress SH0 mode effectively and enhance the amplitude of SH1 mode by 100% in theory and by 25% in the experiment. Moreover, the longitudinal positioning error has more significant influence on the SH0 suppression effect and SH1 enhancement effect than the lateral, so the longitudinal error in the application should be decreased if possible.

## Figures and Tables

**Table 1:** Parameters and values of PPM EMATs in the finite element model.

Object	Parameter	Value
Magnet	Width	3 mm
	Height	5 mm
	Thickness	4 mm
	Gap between adjacent magnets	1 mm
	lift-off distance	0.5 mm
	Magnetic flux density	1.5 T
Coil	Diameter	0.3 mm
	lift-off distance	0.5 mm
	Resistivity	$1.7\times 10^{-8}\;\Omega \cdot m$
Aluminum Plate	Length	50 mm
	Width	15 mm
	Thickness	3 mm
	Density	2700 kg/m
	Conductivity	$3.5\times 10^{-7}\;S/m$
	Young’s modulus	70 Gpa
	Poisson’s ratio	0.33
Excitation current	Amplitude	1 A
	Frequency	700 kHz

**Table 2:** Parameters and values of PPM EMATs in the experiment.

Object	Parameter	Value
Periodic Magnet Array	Width	25 mm
	Height	25 mm
	Thickness	3 mm
	Gap between adjacent magnets	1 mm
	Lift-off distance	0.3 mm
	Magnetic flux density	250 mT
	Number of magnets in each periodic magnet array	14
Racetrack Coil	Width	30 mm
	Length	60 mm
	Thickness	0.1 mm
	Copper layer width	0.7 mm
	Copper layer depth	0.035 mm
	Copper layer interval	0.9 mm
	Turns of each coil	13
	Lift-off distance	0.1 mm
Aluminum Plate	Length	500 mm
	Width	500 mm
	Thickness	3 mm
Excitation Signal	Voltage	260 V
	Operation Frequency	700 kHz
	Duty Ratio	20%
	Repetition Frequency	50 Hz
